# Early-life overconsumption of a high-carbohydrate diet induces metaflammation and kidney injury without impairment of function in adult Wistar rats

**DOI:** 10.1007/s13105-026-01226-7

**Published:** 2026-08-24

**Authors:** Cristhian Neftaly Sánchez-Solís, Hugo Hernández-Fragoso, Alfonso Diaz, Rubén Vázquez-Roque, Olivier Christophe Barbier, José Everardo Avelino-Cruz, Samuel Treviño

**Affiliations:** 1 Laboratory of Metabolomic and Chronic Degenerative Diseases, Physiology Institute, Meritorious Autonomous University of Puebla, Prol. de la 14 Sur 6301, Ciudad Universitaria, Puebla, C.P. 72570 Mexico; 2 Laboratory of Neurochemistry and Behavior, Physiology Institute, Meritorious Autonomous University of Puebla, Prol. de la 14 Sur 6301, Ciudad Universitaria, Puebla, C.P. 72570 Mexico; 3 Laboratory of Neuroplasticity and Metabolism, Physiology Institute, Meritorious Autonomous University of Puebla, Prol. de la 14 Sur 6301, Ciudad Universitaria, Puebla, C.P. 72570 Mexico; 4 Department of Toxicology, Center for Research and Advanced Studies (Cinvestav), México, Mexico; 5 Laboratory of Molecular Cardiology, Institute of Physiology, Meritorious Autonomous University of Puebla, Prol. de la 14 Sur 6301, Ciudad Universitaria, Puebla, C.P. 72570 Mexico

**Keywords:** Overnutrition, Metaflammation, Cytokines, Chronic Kidney Disease

## Abstract

Frequent consumption of sugary and calorie-dense foods and beverages during childhood may contribute to the development of metabolic syndrome (MetS) in adulthood, a risk that can manifest independently of overweight status. Metaflammation, a low-grade inflammation associated with MetS, can induce functional loss in multiple tissues, including the kidneys, potentially signaling the onset of damage. This work aimed to evaluate the progressive structural changes, damage, and kidney function associated with metaflammation-induced MetS in young to adult Wistar rats. Early-life rats were divided into two groups: one fed a standard diet and the other a high-carbohydrate diet (HCD). Three analysis time points were designed: 6 weeks (adolescence), 12 weeks (young adulthood), and 20 weeks (adults). Zoometry, nutritional status, metabolic biomarkers, kidney function, serum and urinary cytokines, and kidney injury molecule 1 (KIM-1) were measured in each cohort. Histopathologic modifications, inflammation, and redox balance were analyzed in the renal cortex. Results show that HCD consumption progressively induces MetS and metaflammation, increasing serum levels of inflammatory cytokines and KIM-1. However, renal function was not affected. Renal cortex inflammation began in adolescence, even before oxidative stress was established in young and adult rats. The renal tissue showed hallmarks of early kidney damage, including mesangial cell expansion, podocyte loss, hypertrophy, tubular hyperplasia, and fibrosis. In urine, levels of KIM-1, TGF-β, and IL-1β increased. In conclusion, chronic HCD consumption from early life establishes MetS and metaflammation, leading to silent nephropathy. This state is characterized by structural remodeling and profibrotic signaling that occur independently of standard clinical kidney function markers in Wistar rats.

## Introduction

Dietary overnutrition refers to excessive intake of energy and nutrients, leading to metabolic imbalance and increased health risks. It refers to deficiencies, excesses, or imbalances in a person’s energy and/or nutrient intake, which lead to an imbalance between energy storage and breakdown [1]. The World Health Organization (WHO) aims for a world free of all forms of malnutrition, including dietary overnutrition. According to the 2016–2025 nutrition strategy, the WHO works to provide access to adequate nutrition and healthy food. However, dietary overnutrition is a globally increasing concern that affects a considerable proportion of children, adolescents, and adults [2]. In Mexico, childhood overweight and obesity have risen steadily in the last three decades, associated with increased consumption of sugary and high-calorie-dense foods and beverages, as well as sedentary lifestyles in obesogenic food environments. The Mexican National Health and Nutrition Survey (ENSANUT) has estimated a high prevalence of overweight and obesity, where 75% of adults and 35% of children and adolescents aged 5–19 years have excess weight, and 6.3% of young children under the age of 5 are overweight [3]. These figures tend to be higher in urban settings.

Overnutrition and obesity are commonly linked by the most widely used indicator of excess body weight, body mass index (BMI). However, BMI has recently been questioned because it does not provide reliable information in all cases about the morpho-functional state of adipose tissue, which stores energy reserves and then poses a high risk of developing metabolic abnormalities [4]. In this way, it has been reported that around 20–40% of subjects with normal body weight have been identified as having metabolic abnormalities. Meanwhile, 20–30% of overweight subjects appear metabolically healthy [5]. Metabolic abnormalities due to dietary overnutrition of high-fat and/or high-carbohydrate diets are observed in altered plasma levels of these molecules in both young and adult subjects (dyslipidemia and dysglycemia), as well as in hormone concentrations such as insulin, leptin, and adiponectin, which are also impaired. It has been reported that in chronic endocrine-metabolic abnormalities (e.g., MetS), inflammatory mediators emerge and antagonize the homeostatic systems that regulate lipids, glucose, and hormones, thereby generating a vicious cycle. Metabolically disease-associated inflammation is referred to as meta-inflammation or metaflammation, and it can be detected in numerous tissues [6].

Metaflammation is a low-grade, chronic, non-resolving inflammatory response characterized by the activation of pro-inflammatory cytokines, such as tumor necrosis factor-alpha (TNF-α), interleukin 1 beta (IL-1β), and IL-6, which accelerate metabolic alterations, while anti-inflammatory cytokines are depleted. Metaflammation recruits M1 macrophages, while reducing M2 macrophages, thereby establishing a state of inflammaging across multiple tissues [6]. Interestingly, little work links MetS, metaflammation, and chronic kidney disease. Kidneys receive a quarter of cardiac output; thus, in subjects with MetS and metaflammation, they handle elevated levels of lipoproteins, glucose, and hormones to maintain homeostasis, but are also exposed to high fluxes of systemic pro-inflammatory mediators and oxidized metabolic byproducts. However, the kidneys do not possess the same levels of antioxidant, detoxifying, and anti-inflammatory defense mechanisms as other highly vascularized tissues, such as hepatic tissue. Hence, the kidney is a vulnerable target of persistent aggression, which can result in damage or loss of function [7]. Therefore, renal metabolic stress is often an overlooked component of the MetS cluster, which can progress to chronic disease or silent nephropathy. This structural damage precedes detectable changes in clinical markers of function, such as GFR and proteinuria.

Chronic kidney disease (CKD) is defined as “abnormalities of the kidney structure or function, present for more than 3 months, with health implications” [8]. In the early stages, silent nephropathy, renal adaptations in response to MetS include hypertrophy or hyperplasia of glomeruli and tubules, and there is no doubt that inflammation plays a critical role in the progression and outcome of renal damage [9]. However, the link between disease initiation and inflammation remains under debate. We hypothesize that the chronic consumption of a high-carbohydrate diet in young Wistar rats triggers a state of systemic metaflammation that progressively induces MetS, leading to time-dependent early stages of kidney disease, and a shift in the urinary pro-inflammatory/pro-fibrotic cytokine profile as the animals transition from youth to adulthood. Therefore, this work aimed to evaluate the progressive structural changes, damage, and kidney function associated with metaflammation-induced MetS in young to adult Wistar rats.

## Materials and methods

### Animals and diets

From the “Claude Bernard” vivarium of the Autonomous University of Puebla, sixty male Wistar rats (one month old and 70–100 g) were provided. The animals were kept at a regulated temperature (22 °C), with a 12-hour light:12-hour dark cycle, and had free access to food and water. Rats were randomly assigned to two groups. The normocaloric group (NCD = 30) was fed a standard chow diet (5001, LabDiet; St. Louis, MO, USA), and the hypercaloric group (HCD = 30), which was provided with a high carbohydrate diet (Based on the patent: MX/E/2013/047377, to generate the model of MetS) by 6-, 12-, and 20-weeks, to simulate feeding from early age to adulthood. The procedures described in this study were performed following the Standards for the Use and Care of Laboratory Animals described by the Mexican Council for Animal Care (NOM-062-ZOO-1999), as well as the Guide of the National Institutes of Health for the Care and Use of Laboratory Animals, and the Ethics Committee of the Autonomous University of Puebla (Approval by CICUAL No. VIEP/DGI/0350/2024; February 27th, 2024). All applicable international (ARRIVE and NHI guidelines), national, and institutional guidelines for the care and use of animals were followed to minimize possible discomfort.

Only male rats were included in this study to avoid the influence of cyclical hormonal fluctuations on cytokine expression and renal hemodynamics, as well as to maintain consistency with previous experimental models of diet-induced metabolic syndrome in Wistar rats.

### Zoometric parameters and nutritional status

The weight of the rats was determined using a digital scale (Torrey, model LPCR-20/40, Querétaro, Mexico), and size was measured weekly from the base of the tail to the tip of the nose. As previously reported, weight gain, body mass index (BMI), and Lee index were calculated [9]. Food and water consumption were assessed daily to determine the caloric intake by each experimental group. The visceral adipose index (VAI) was calculated using a previously reported formula incorporating abdominal circumference, BMI, triglycerides, and cholesterol in high-density lipoprotein (HDL-C) concentration [4, 10]. The dysfunctional adiposity index (DAI), an early marker of adipocyte morpho-functional abnormalities, was calculated as mentioned by [5]. Additionally, adipocyte function was analyzed by the adiponectin/leptin ratio [6]. Animals were allocated 2 days per week to individual metabolic cages for 24 h. This protocol enabled habituation and monitoring of food and water intake, body weight fluctuations, and stress- and inflammation-related biomarkers to establish a stable baseline. Environmental conditions during this period were maintained identically to the vivarium settings with *ad libitum* access to water and their designated diets. Diet intake was monitored, and the weight of feed consumed (Wd) was calculated as Wd = W0 (initial food weight) − W1 (leftover food weight) − W2 (spilled food weight). The daily energy intake per rat (kcal/day/rat) was calculated as Wd × the food’s energy content (kcal/g). The nutrient content in each diet was reported previously [9].

### Serum and urinary biochemical parameters

Rats were housed in metabolic cages for 24 h, two days before sacrifice, to collect urine. The urinary volume was measured, and then the sample was centrifuged (400 × g for 3 min). The supernatant was separated and frozen at − 70 °C until analysis. Commercial kits were used to quantify urinary urea and creatinine in an automated spectrophotometer (A-15 BioSystems, Guadalajara, Mexico). Albumin concentration in urine was quantified using an immunofluorescence analyzer (ichromaTM II, Boditech Med Inc., Korea). The albumin/creatinine ratio was calculated and expressed as mg/g. Creatinine clearance was calculated as follows: ([urinary creatinine × (volume in mL/1440)]/serum creatinine) × (body surface); the body surface was calculated according to Sánchez-Solís et al. [9]. Then, animals were fasted for 4–5 h before sacrifice and anesthetized intraperitoneally with a dose of Ketamine + Xylazine 0.2 mL/100 g of body weight. Blood samples were obtained by intracardiac puncture, collected in BD Vacutainer^®^ SST™ Tubes (Becton, Dickinson and Company, USA), and centrifuged (800 × g/10 min). The serum was separated and frozen at − 70 °C until analysis. Glucose, fructosamine, triglycerides (TG), total cholesterol (TC), cholesterol in low-density lipoprotein (LDL-C), HDL-C, urea, and creatinine levels were quantified by commercial kits in an automated spectrophotometer (A-15 BioSystems, Guadalajara, Mex.). Very low-density lipoprotein (VLDL) levels were obtained using the Martin-Hopkins estimation [11]. As previously reported, the free fatty acid concentration (FFA) was determined [6]. The concentrations of insulin, adiponectin, and leptin were measured using ELISA immunoassay kits (Diagnostica Internacional Company, Guadalajara, Mexico) in a Stat Fax 2600 plate reader at 415 nm (WinerLab Group, Buenos Aires, Argentina). The HOMA-IR index was calculated as reported by Treviño et al. [12]. For euthanasia, an overdose of sodium pentobarbital (60 mg/kg; i.p.) was used, which acts as a CNS depressant, leading to respiratory and cardiac arrest. It was used because it minimizes animal distress and maintains tissue integrity for histological and molecular analysis. The kidneys were removed and sectioned along the longitudinal coronal plane; the cortex from the right kidney was quickly separated and placed in ultracongelation at −70 °C for quantification of redox balance (*n* = 10 per group and each time point analyzed). The left kidney sections were immersed in neutral buffered formalin (4%), one half for histology and the other for immunofluorescence tests (*n* = 10 per group and per time point analyzed).

### Quantification of cytokines in serum and urine

Cytokine concentrations in serum and urine were quantified using a sandwich immunoassay. 20 µL of the sample was preincubated with 100 µL carbonate/bicarbonate buffer (pH = 9.6) in the wells for 24 h at 4 °C. Subsequently, the wells were washed with PBS-Tween 20, and the nonspecific binding sites were blocked with 2% bovine serum albumin (BSA; Sigma-Aldrich; Toluca, Mexico) for 2 h at 4 °C. The samples were incubated with the primary antibodies: IL-6, TNF-α, IL-1β, IL-10 (1:500; Santa Cruz Biotechnology, USA), transforming growth factor beta (TGF-β), and kidney injury molecule 1 (KIM-1) (1:500; Cell Signaling Technology, USA), and secondary antibody conjugated with a horseradish peroxidase were used (1:1000, Jackson Immuno Research Laboratories and Abcam, USA). After washing, 200 µL of DMAB/MBTH substrate solution was added to each well, and the wells were incubated for 7 min at room temperature. The enzyme reaction yielded a blue product, which turned yellow upon addition of the stop solution (R&D Systems, Minneapolis, MN). The measured color intensity was proportional to the amount of cytokine. Samples were read at 450 nm with a SmartSpec 3000 spectrophotometer (BioRad, Hercules, CA, USA). A standard curve for each cytokine was generated (0–1000 pg/mL) with a low limit of detection of 0.1–1.5 pg/mL, and a correlation coefficient (concentration/absorbance) of *r* = 0.995. Cytokine urinary concentrations were expressed as picograms per milligram of urine creatinine.

### Renal histology analysis

After 24 h of fixation, coronal sections (*n* = 10 per group, with analysis at each time point) were embedded in paraffin in an automated Leica TP1020 Semi-Enclosed Tissue Processor (Leica Biosystems, Nussloch, Germany), and then 5 μm-thick sections were cut on a Leica RM2125 RTS rotary microtome (Leica Biosystems, Nussloch, Germany). Slides were stained (2–4 per rat) with Periodic Acid- Schiff (PAS) and Masson’s trichrome (MT). To ensure anatomical consistency across all animals and experimental groups, evaluations were strictly confined to the renal cortex. A total of 250 glomeruli were randomly selected per group. The following parameters were analyzed: glomerular area, glomerular tuft area, Bowman’s space area, sclerotic index, and the number of parietal cells, mesangial cells, and podocytes. Additionally, 250 proximal tubules were analyzed for the tubular-to-luminal area ratio and the number of cells per tubule. Proximal convoluted tubules were identified within the renal cortex based on distinct histomorphological and staining criteria, specifically: their localization surrounding renal corpuscles, a simple cuboidal epithelium with large central nuclei, a larger outer diameter with a characteristically narrow/occluded lumen, and the presence of a prominent, intensely PAS-positive brush border on the apical membrane. Distal tubules and collecting ducts, which lack a prominent brush border and exhibit a clearer, wider lumen, were excluded from this analysis. To ensure the highest level of objectivity, a triple-blind design was implemented. Histotechnologists performed tissue processing, embedding, and staining using randomized alphanumeric codes, effectively masking the diet and time-point assignments. These de-identified slides were subsequently evaluated by a pathologist who remained blinded to the experimental groups until histomorphometric scoring was completed. Photomicrographs from the renal cortex were taken at 40X magnification using a Microscope Axio Imager.A2m and an AxioCam ERc5s camera and image processing program ZEN2011 (Carl Zeiss Microscopy, White Plains, NY, USA). All measurements were carried out using the Motic MIplus program (Motic China Group Co., Ltd., Xiamen, China) and ImageJ software version 1.54 (National Institutes of Health, Bethesda, MD, USA). A semiquantitative score (sclerosis index) was used to assess the extent and severity of glomerular sclerosis. The severity of sclerosis for each glomerulus was graded from 0 to 4 as follows: 0 represents no lesion, 1 represents sclerosis of < 25% of the glomerulus, while 2, 3, and 4 represent sclerosis of 25% to 50%, 50% to 75%, and > 75% of the glomerulus, respectively. A whole-kidney average sclerosis index was obtained by averaging scores across all glomeruli in a single section. All histological evaluations and quantitative morphometric measurements were performed by an investigator blinded to the experimental groups, under the supervision of the principal researcher and an experienced pathologist.

### Immunofluorescence

Coronal Sect. (5 μm) were deparaffinized and rehydrated (2 slides per mark from 10 kidneys per group), with analysis at each time point to analyze 250 glomeruli and 250 proximal convoluted tubules. Afterward, antigenic recovery was performed in Diva Decloaker 20X (Biocare Medical, CdMX, Mex) for 40 min at 60 °C. The samples were incubated with BSA (2%, Sigma-Aldrich; Toluca, Mex.) for blocking for 2 hours at 4 °C. Subsequently, tissue sections were incubated with a primary antibody IL-6, TNF-α, IL-1β, IL-10 (1:400; Santa Cruz Biotechnology, USA), TGF-β, and KIM-1 (1:400; Cell Signaling Technology, USA) overnight at 4 °C, successively by antirabbit rhodamine-labeled or anti-mouse FITC-labeled secondary antibodies (1:400, Jackson Immuno Research Laboratories, USA). The tissues were counterstained with VECTASHIELD (Vector Laboratories, USA). Photomicrographs were obtained from the renal cortex at 40X magnification using a fluorescence microscope (Leica Microsystems, Wetzlar, Germany). Glomeruli and tubules were analyzed using ImageJ software version 1.54 (National Institutes of Health, Bethesda, MD, USA), and data were reported as the density in arbitrary units (Pixels). Images were spatially calibrated by converting pixel dimensions to micrometers using a stage micrometer corresponding to the objective’s magnification. The multi-channel color images were split into individual grayscale channels (8-bit format). In this format, each pixel is assigned a digital number (gray value) ranging from 0 (pure black/no signal) to 255 (pure white/maximum intensity saturation). To quantify specific structural expression, regions of interest (such as the renal cortex, glomerular tuft, or tubular boundaries) were manually outlined using the Freehand selection tool. The software calculated the integrated density (the sum of the gray values of all pixels within the selected area) and the total area of the ROI (Region of Interest in pixels^2^ or µm^2^). The Mean Fluorescence Intensity (MFI), representing the actual fluorescence density, was determined using the formula: MFI = Integrated density/area of ROI. To account for non-specific background glare, the MFI of an adjacent, unlabeled tissue region was subtracted from each measurement to yield the corrected total fluorescence (CTF). A total of 250 random fields (glomeruli or tubules) per section were analyzed to ensure a representative and unbiased quantification of cytokine expression across all experimental groups.

### Redox balance

Reactive oxygen species (ROS) were measured using the 2′,7′-dichlorodihydrofluorescein diacetate (DCFH-DA; Sigma-Aldrich; Toluca, Mexico) method in a PerkinElmer LS50-B luminescence spectrometer (Waltham, MA, USA); results were expressed as micromoles per milligram of protein. Nitrites were measured using the Griess reaction (Sigma-Aldrich; Toluca, Mexico) in a SmartSpec 3000 spectrophotometer (Bio-Rad, Hercules, CA, USA); the results were expressed as µM/mg of protein. The concentration of malondialdehyde (MDA) and 4-hydroxyalkenals (4HDA) was quantified by colorimetric reaction using N-methyl-2-phenyl-indole in a mixture of acetonitrile and methanol (3:1) and 150 µL of methanesulfonic acid or hydrochloric acid (Sigma-Aldrich; Toluca, Mexico) in a SmartSpec 3000 spectrophotometer (Bio-Rad, Hercules, CA, USA); the results were expressed as µM/mg of protein [13–15].

The total glutathione (GSH), GSH, and GSSG concentrations were measured using enzymatic recycling methods (Sigma-Aldrich, Toluca, Mexico) on a PerkinElmer Lambda EZ-150 spectrophotometer (Waltham, MA, USA); the results were expressed as µM/mg protein. Glutathione peroxidase (GPx; nmol min^− 1^/mg of protein), glutathione S-transferase (GST; U min^− 1^/mg of protein), and glutathione reductase (GR; mU min^− 1^/mg of protein) activities were measured. Catalase (CAT; U min^− 1^/mg protein) and superoxide dismutase (SOD; U min^− 1^/mg protein) activities were quantified using the Aebi and auto-oxidized pyrogallol methodologies, respectively. Enzymatic activities were analyzed using Sigma-Aldrich reagents (Toluca, Mexico) in a PerkinElmer Lambda EZ-150 spectrophotometer (Waltham, MA, USA). Protocols are described in more detail, as previously reported [13–15].

### Statistics

The results were expressed as the mean ± SEM for all experiments. Normality was assessed using the Shapiro-Wilk test. Zoometric, energy intake, serum, and urinary parameters were analyzed using a parametric Two-Way Repeated Measures ANOVA to evaluate the effect of a high-carbohydrate diet versus time on consumption, followed by Bonferroni’s post hoc test. Data analysis was performed with GraphPad Prism 8 (GraphPad Software Inc., USA). Values of *P* ≤ 0.05 were considered statistically significant. Meanwhile, histological parameters were analyzed using a nonparametric analysis of variance of aligned rank-transformed (ART) data, followed by a post hoc test using Turkey’s method; *P* ≤ 0.05 was considered significant. RStudio (Version 1.0.143) and the Statistical Package for the Social Sciences (IBM SPSS Version 21, SPSS Inc., 2009) were used for data analysis.

## Results

### Chronic consumption of a high-carbohydrate diet alters nutritional status, zoometric parameters, and the biochemical serum profile

A two-way repeated-measures ANOVA was conducted to examine the effects of diet (NCD vs. HCD) and time (6, 12, and 20 weeks) on nutritional status, zoometric parameters, and the biochemical serum profile. Despite HCD groups increasing their food intake (31–33%) and daily caloric intake (25–39%; Table 1), they did not increase body weight or BMI; the interaction between variables was highly significant (F_(5, 45)_ = 36.74, *P* < 0.0001 and F_(5, 45)_ = 6.88, *P* < 0.0001). Meanwhile, adiposity, as measured by the Lee index, increased to 6 weeks (20%) and then decreased by 14.2% at 20 weeks, but the interaction was highly significant (F_(5, 45)_ = 35.48, *P* < 0.0001). However, VAI and DAI increased by 31% to 98% after 6 weeks of hypercaloric consumption, indicating adipocyte dysfunction, as confirmed by the adiponectin-leptin ratio, which decreased by 37% to 55% after 12 weeks of HCD (F_(5, 45)_ = 18.25, *P* < 0.0001, F_(5, 45)_ = 16.9, *P* < 0.0001, and F_(5, 45)_ = 11.21, *P* < 0.001). Visceral adipocyte dysfunction indicates metabolic dysfunction. Therefore, we analyzed biochemical parameters. The HCD groups showed a time-dependent increase in fasting glucose (45%, 56%, and 84%; F_(5, 45)_ = 25.65, *P* < 0.0001), fructosamine (54%, 37%, and 45%; F_(5, 45)_ = 26.56, *P* < 0.0001), and hyperinsulinemia, resulting in insulin resistance (HOMA-IR: 58%, 172%, and 385%; F_(5, 45)_ = 48.87, *P* < 0.0001). The lipid profile was also altered, with increases in triglycerides (F_(5, 45)_ = 15.43, *P* < 0.0001), total cholesterol (F_(5, 45)_ = 46.62, *P* < 0.0001), VLDL-C (F_(5, 45)_ = 13.4, *P* < 0.0001), LDL-C (F_(5, 45)_ = 24.3, *P* < 0.0001), and free fatty acids (F_(5, 45)_ = 78.75, *P* < 0.0001), while HDL-C progressively decreased (F_(5, 45)_ = 65.19, *P* < 0.0001). The statistical analysis confirmed that chronic HCD consumption has a stronger interaction with time in inducing MetS (Table 1), as observed in humans.

**Table 1:** Nutritional status, zoometric parameters, and biochemical serum profile

	6 weeks			12 weeks			20 weeks		
	NCD *n* = 10		HCD *n* = 10	NCD *n* = 10		HCD *n* = 10	NCD *n* = 10		HCD *n* = 10
Zoometric profile									
Body weight (g)	211.7 ± 10.5		180 ± 8.19	235.8 ± 14.1		209.3 ± 3.6	309.6 ± 11		256 ± 10.4▼
BMI (g/cm^2^)	0.66 ± 0.01		0.6 ± 0.02	0.67 ± 0.01		0.67 ± 0.01	0.74 ± 0.01		0.65 ± 0.01▼
Lee index (%)	4.44 ± 0.16		5.33 ± 0.13▲	4.85 ± 0.09		4.73 ± 0.08	5.67 ± 0.08		4.86 ± 0.12▼
VAI	0.74 ± 0.02		0.98 ± 0.1▲	1.28 ± 0.12		2.31 ± 0.13▲	1.14 ± 0.11		2.26 ± 0.13▲
DAI	0.84 ± 0.02		1.1 ± 0.11▲	1.46 ± 0.16		2.63 ± 0.14▲	1.30 ± 0.12		2.56 ± 0.15▲
Adipocyte function	48.5 ± 8.8		39.1 ± 9.5	46.0 ± 3.6		29.05 ± 2.6▼	47.3 ± 6.2		21.3 ± 4.9▼
Food intake									
Food consumption (g/day)	21.1 ± 0.33		27.6 ± 1.04▲	20.1 ± 0.48		33.7 ± 0.62▲	26.9 ± 0.88		35.5 ± 0.60▲
Total energy (Kcal/day)	71 ± 1.11		97.4 ± 3.62▲	67.6 ± 1.64		118 ± 2.24▲	90.5 ± 3.0		126 ± 2.16▲
Glycemic profile									
Fasting glucose (mg/dL)	99 ± 1.97		104 ± 8.72	96.4 ± 5.89		150 ± 6.06▲	92.4 ± 5.63		170 ± 7.03▲
Fructosamine (mmol/L)	21.5 ± 2.2		33.2 ± 2.13▲	27.7 ± 2.69		38.1 ± 2▲	35.9 ± 2.1		52.1 ± 2.5▲
Fasting insulin (µUI/mL)	9.85 ± 0.07		10.7 ± 0.69	11.1 ± 0.69		20.1 ± 1.42▲	11 ± 0.44		29.2 ± 0.33▲
HOMA-IR	0.40 ± 0.01		0.46 ± 0.03	0.44 ± 0.04		1.24 ± 0.09▲	0.42 ± 0.02		2.04 ± 0.03▲
Lipid profile									
Triglycerides (mg/dL)	73 ± 2.41		93.2 ± 4.9▲	86 ± 5.25		123 ± 3.83▲	82.3 ± 5.1		118 ± 5.96▲
Total cholesterol (mg/dL)	91.9 ± 1.4		112.7 ± 2.14▲	98.4 ± 3.84		104.7 ± 2.47	96.3 ± 2.9		115.1 ± 2.8▲
VLDL-C (mg/dL)	15.3 ± 0.96		21.7 ± 1.02▲	20.7 ± 0.93		17.1 ± 0.29	10.8 ± 1.5		16.9 ± 0.94▲
LDL-C (mg/dL)	31.7 ± 1.40		46.6 ± 2.6▲	34 ± 4.55		54 ± 2.92▲	37.1 ± 2.8		64.7 ± 5.42▲
HDL-C (mg/dL)	44.9 ± 1.69		44.4 ± 1.23	43.7 ± 4.16		33.6 ± 1.65▼	48.4 ± 3.59		33.5 ± 1.99▼
FFA (mg/dL)	3.62 ± 0.77		5.73 ± 0.25▲	1.83 ± 0.22		3.86 ± 0.45▲	6.50 ± 0.49		34.6 ± 3.23▲

The results shown are the average of 10 different experiments ± SEM. A Two-Way Repeated Measures ANOVA followed by Bonferroni’s post hoc test with a significance level set at *p* ≤ 0.05 was performed. (▲) indicates a significant difference with values above those of the control group. (▼) indicates a significant difference with values below those of the control group. *BMI* Body mass index, *HOMA-IR* Homeostatic model assessment for insulin resistance, *VLDL-C* Very low-density lipoprotein, *LDL-C* Low-density lipoprotein, *HDL-C* High-density lipoprotein, *FFA* Free fatty acids

### Metabolic syndrome is associated with low-grade inflammation in the peripheral and kidney

**Fig. 1:**
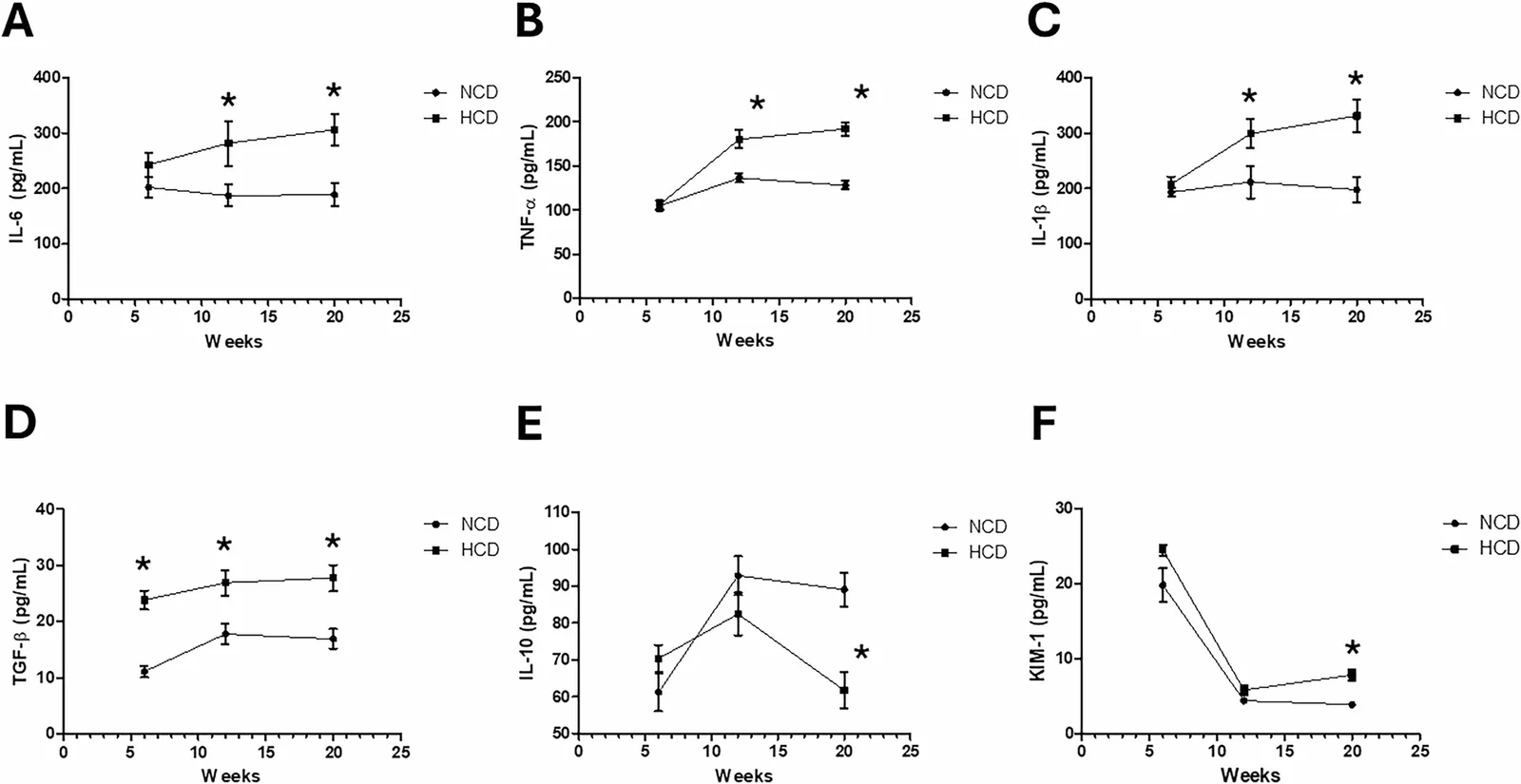
Serum cytokines and KIM-1. NCD is the standard diet group; HCD is the high-carbohydrate diet group. The results shown are the average of ten different experiments ± SEM. A Two-Way Repeated Measures ANOVA followed by Bonferroni’s post hoc test with a significance level of *P* ≤ 0.05 vs. control was performed. The symbol (*) indicates a significant difference compared to the control group. IL-6, interleukin 6; TNF-α, tumor necrosis factor-alpha; IL-1β, interleukin 1 beta; TGF-β, transforming growth factor beta; IL-10, interleukin 10; KIM-1, kidney injury molecule 1

The metabolic syndrome induces low-grade inflammation; therefore, we measured serum pro-inflammatory cytokines. The HCD groups showed increases in serum concentrations of IL-6 (50% and 61%; F_(5, 45)_ = 3.87, *P* = 0.0053), TNF-α (32% and 49%; F_(5, 45)_ = 31.96, *P* < 0.0001), and IL-1β (41% and 82%; F_(5, 45)_ = 5.96, *P* < 0.0001) from 12 weeks. Likewise, anti-inflammatory cytokines, such as TGF-β, also increased by 114%, 60%, and 74% from 6 weeks of HCD consumption (F_(5, 45)_ = 11.88, *P* < 0.0001). Meanwhile, IL-10 serum concentration diminished by 30.6% after 20 weeks in the HCD group (F_(5, 45)_ = 8.56, *P* < 0.0001). We also quantified KIM-1 serum levels, a marker of renal damage, which increased by 101% over 20 weeks (F_(5, 45)_ = 97.16, *P* < 0.0001; Fig. 1A-F).

**Fig. 2:**
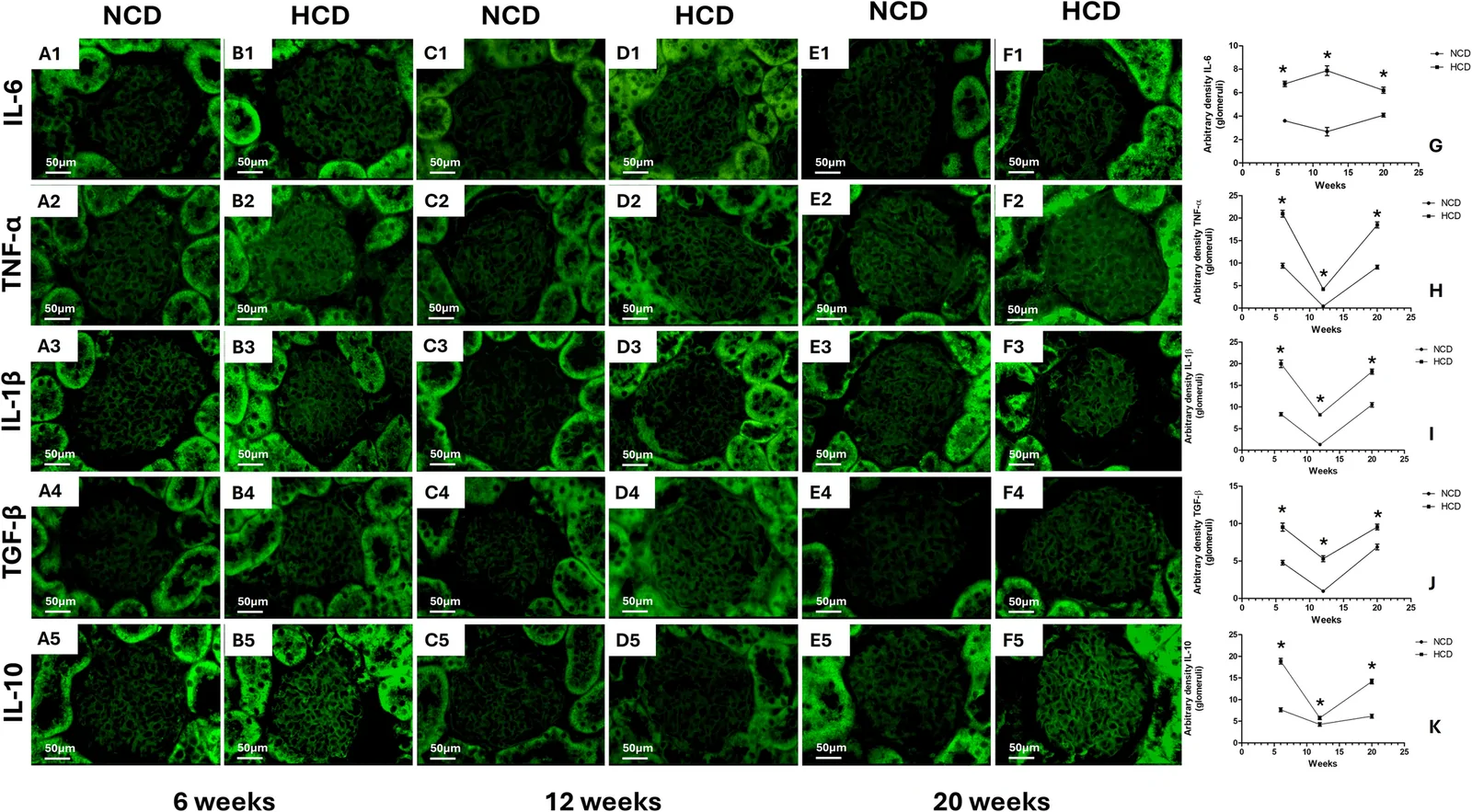
Glomerular cytokine immunoreactivity. NCD is the standard diet group; HCD is the high-carbohydrate diet group. The results shown are the average immunoreactivity of 250 glomeruli ± SEM. A nonparametric analysis of variance of aligned rank-transformed (ART) data, followed by a post hoc test using Turkey’s method, was performed at the *P* ≤ 0.05 significance level vs. control. The symbol (*) indicates a significant difference compared with the control group. IL-6, interleukin 6; TNF-α, tumor necrosis factor-alpha; IL-1β, interleukin 1 beta; TGF-β, transforming growth factor beta; IL-10, interleukin 10

The kidney’s inflammatory status was analyzed by immunoreactivity (green). Pro- and anti-inflammatory cytokines were evaluated at the glomerular and tubular levels. After studying the delimited glomerular area, IL-6 immunoreactivity increased by 87%, 195%, and 151% in the HCD groups (F_(5, 245)_ = 31.14, *P* < 0.0001; Fig. 2A1-F1 and G), TNF-α by 122%, 900%, and 103% (F_(5, 245)_ = 11.19, *P* < 0.0001; Fig. 2A2-F2 and H), and IL-1β by 140%, 624%, and 73% (F_(5, 245)_ = 0.355, *P* = 0.161; Fig. 2A3-F3 and I). At the same time, in the HCD groups, the anti-inflammatory response also increased, with TGF-β levels rising by 99%, 540%, and 138% (F_(5, 245)_ = 9.87, *P* < 0.0001; Fig. 2A4-F4 and J), while IL-10 levels increased by 148%, 37%, and 232% (F_(5, 245)_ = 14.92, *P* < 0.0001; Fig. 2A5-F5 and K). The ART ANOVA confirms that both HCD and time matter, but they matter together. The HCD diet significantly increases glomerular inflammation and early pro-fibrosis at (6–12 weeks). After week 12, the HCD group observed the process stabilizing.

**Fig. 3:**
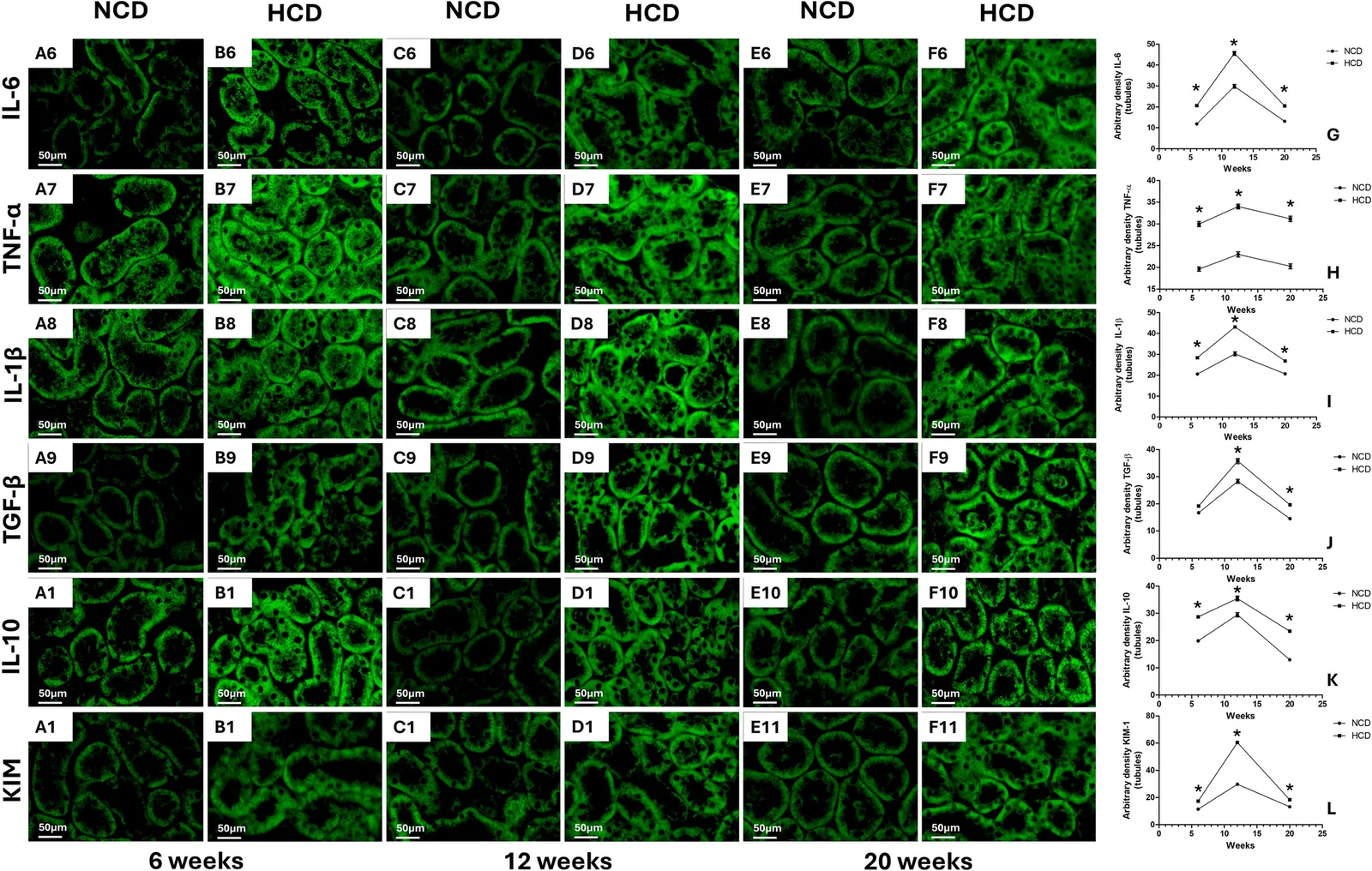
Cortex renal tubules cytokine and KIM-1 immunoreactivity. NCD is the standard diet group; HCD is the high-carbohydrate diet group. The results shown are the average immunoreactivity of 250 tubules ± SEM. A nonparametric analysis of variance of aligned rank-transformed (ART) data, followed by a post hoc test using Turkey’s method, was performed at the *P* ≤ 0.05 significance level vs. control. The symbol (*) indicates a significant difference compared with the control group. IL-6, interleukin 6; TNF-α, tumor necrosis factor-alpha; IL-1β, interleukin 1 beta; TGF-β, transforming growth factor beta; IL-10, interleukin 10; KIM-1, kidney injury molecule 1

Similar results were observed at the tubular level. A tubular structure showed high immunoreactivity for IL-6 (73%, 52%, and 56%; F_(5, 245)_ = 92.04, *P* < 0.0001; Fig. 3A6-F6 and G), TNF-α (52%, 47%, and 53%; F_(5, 245)_ = 19.34, *P* < 0.0001; Fig. 3A7-F7 and H), and IL-1β (37%, 42%, and 29%; F_(5, 245)_ = 16.82, *P* < 0.0001; Fig. 3A8-F8 and I) in the HCD groups compared to the control groups. The ART analysis confirms that in tubules, the HCD has a profound impact on inflammation that compounds over time. While the control group (NCD) remains relatively stable (with a minor increase at week 20), the HCD group exhibits a sharp, non-linear increase, particularly between weeks 12 and 20, indicating that the difference between NCD and HCD is not static; it significantly expands as time progresses from 6 to 20 weeks. Anti-inflammatory cytokines also augmented TGF-β by 14%, 26%, and 34% (F_(5, 245)_ = 22.18, *P* < 0.0001; Fig. 3A9-F9 and J), and IL-10 by 44%, 20%, and 80% (F_(5, 245)_ = 24.82, *P* < 0.0001; Fig. 3A10-F10 and K) at the analyzed cohort times. Additionally, KIM-1 expression in the HCD groups showed increased immunoreactivity at 6 weeks (52%), 12 weeks (102%), and 20 weeks (139%) compared with their control groups (F_(5, 245)_ = 19.34, *P* < 0.0001; Fig. 3A11-F11 and L). The ART analysis confirms that the HCD drives significantly greater effects on pro-fibrotic and kidney-damage markers. Crucially, the interaction analysis shows that the HCD’s physiological impact is cumulative, leading to a dramatic divergence from the control group by week 20.

### Metabolic syndrome impairs renal redox balance

Both metabolic alterations and inflammation can affect renal redox balance (Table 2). Parameters such as ROS increased at 12 and 20 weeks of HCD consumption by 83.3% and 123.6% (F_(5, 45)_ = 21.45, *P* < 0.001), 4-HDA by 84.6% and 301% (F_(5, 45)_ = 28.56, *P* < 0.001), and GSSG by 80.7% and 169% (F_(5, 45)_ = 14.9, *P* < 0.001), while only at 20 weeks of HCD consumption NO_2_^−^ and MDA augmented their renal concentration by 129% 72.4% (F_(5, 45)_ = 15.12, *P* < 0.001 and F_(5, 45)_ = 10.88, *P* < 0.001). Nevertheless, all of them show significant interactions between diet and time. This indicates that the effect of the HCD on these oxidative stress markers becomes progressively more severe over the 20 weeks, as shown by the 2GSH/GSSG index, which diminished by 57.9% and 71.9% (F_(5, 45)_ = 45.12, *P* < 0.0001). Meanwhile, SOD and CAT activities increased at 12 weeks of HCD consumption by 146.8% and 77.8%, and at 20 weeks by 77.6% and 188.5% (F_(5, 45)_ = 19.82, *P* < 0.001; F_(5, 45)_ = 24.15, *P* < 0.001). Notably, the non-enzymatic antioxidant defense, such as GSH, decreased significantly over time by 16.2%, 23.8%, and 24.4% (F_(5, 45)_ = 42.67, *P* < 0.0001) and GPx activity by 60%, 55.2%, and 60% (F_(5, 45)_ = 55.18, *P* < 0.0001), with a significant interaction driven by HCD consumption (Table 2). While GR and GST increased their activity at 20 weeks to counteract rising oxidative stress by 71.4% and 51.4% (F_(5, 45)_ = 10.86, *P* < 0.001 and F_(5, 45)_ = 92.64, *P* < 0.0001).

**Table 2:** Redox balance in the renal cortex

	6 weeks			12 weeks			20 weeks		
	NCD *n* = 10		HCD *n* = 10	NCD *n* = 10		HCD *n* = 10	NCD *n* = 10		HCD *n* = 10
ROS µM/mg of protein	1.22 ± 0.22		0.99 ± 0.3	0.90 ± 0.3		1.65 ± 0.11▲	0.89 ± 0.07		1.99 ± 0.11▲
NO _2_^−^µM/mg of protein	11.3 ± 2.32		4.78 ± 0.57▼	7.51 ± 0.84		7.10 ± 1.19	12.1 ± 3.48		27.7 ± 4.11▲
MDA µM/mg of protein	0.55 ± 0.19		0.75 ± 0.08	0.67 ± 0.01		0.76 ± 0.01	0.98 ± 0.11		1.69 ± 0.05▲
4-HDA µM/mg of protein	4.60 ± 0.97		4.56 ± 1.07	4.92 ± 0.49		9.08 ± 1.25▲	5.33 ± 0.89		21.4 ± 1.69▲
Total Glutathione (µM/mg of protein)	52 ± 4.29		43.4 ± 7.4	57 ± 8.9		44.6 ± 8.8	55.2 ± 5.5		44 ± 13.4
GSH (µM/mg of protein)	50.6 ± 2.8		42.4 ± 1.8▼	55.9 ± 4.5		42.6 ± 4.4▼	54 ± 2.7		40.8 ± 6.8▼
GSSG (µM/mg of protein)	1.4 ± 0.30		1.0 ± 0.13	1.09 ± 0.2		1.97 ± 0.09▲	1.19 ± 0.43		3.2 ± 0.27▲
2GSH/GSSG	72.3 ± 1.7		84.8 ± 2.5▲	102.6 ± 2.3		43.2 ± 2.3▼	90.8 ± 1.7		25.5 ± 3.6▼
GPx nmol min^− 1^/mg of prot	15 ± 1.1		6 ± 2.7▼	12.5 ± 3.7		5.6 ± 2.2▼	11.5 ± 1.4		4.6 ± 1.4▼
GR mU min^− 1^/mg of prot	975.8 ± 21.4		1012 ± 21.4	984 ± 38.4		1047 ± 31.7	963 ± 42.3		1651 ± 36.1▲
GTS U min^− 1^/mg of prot	453 ± 26		517 ± 42	520 ± 36.5		571 ± 49.6	508 ± 31.2		769 ± 21.8▲
SOD U min^− 1^/mg of prot	6.7 ± 0.8		2.9 ± 0.5▼	4.7 ± 0.8		11.6 ± 1.0▲	4.9 ± 0.7		8.7 ± 0.5▲
CAT U min^− 1^/mg of prot	7.8 ± 1.3		4.9 ± 0.7▼	7.2 ± 1.5		12.8 ± 0.7▲	7.8 ± 0.6		22.5 ± 3.9▲

The results shown are the average of 10 different experiments ± SEM. A Two-Way Repeated Measures ANOVA followed by Bonferroni’s post hoc test with a significance level set at *p* ≤ 0.05 was performed. (▲) indicates a significant difference with values above those of the control group. (▼) indicates a significant difference with values below those of the control group. *ROS* Reactive oxygen species, *NO*_2_^−^, Nitrite, *MDA* Malondialdehyde, *4-HDA* 4-hydroxyalkenal, *GSH* reduced glutathione, *GSSG* oxidized glutathione, *2GSH/GSSG* redox index, *GPx* glutathione peroxidase, *GR* glutathione reductase, *GTS* glutathione S-transferase, *SOD* superoxide dismutase, *CAT* catalase

### Metabolic syndrome modifies renal histology

**Fig. 4:**
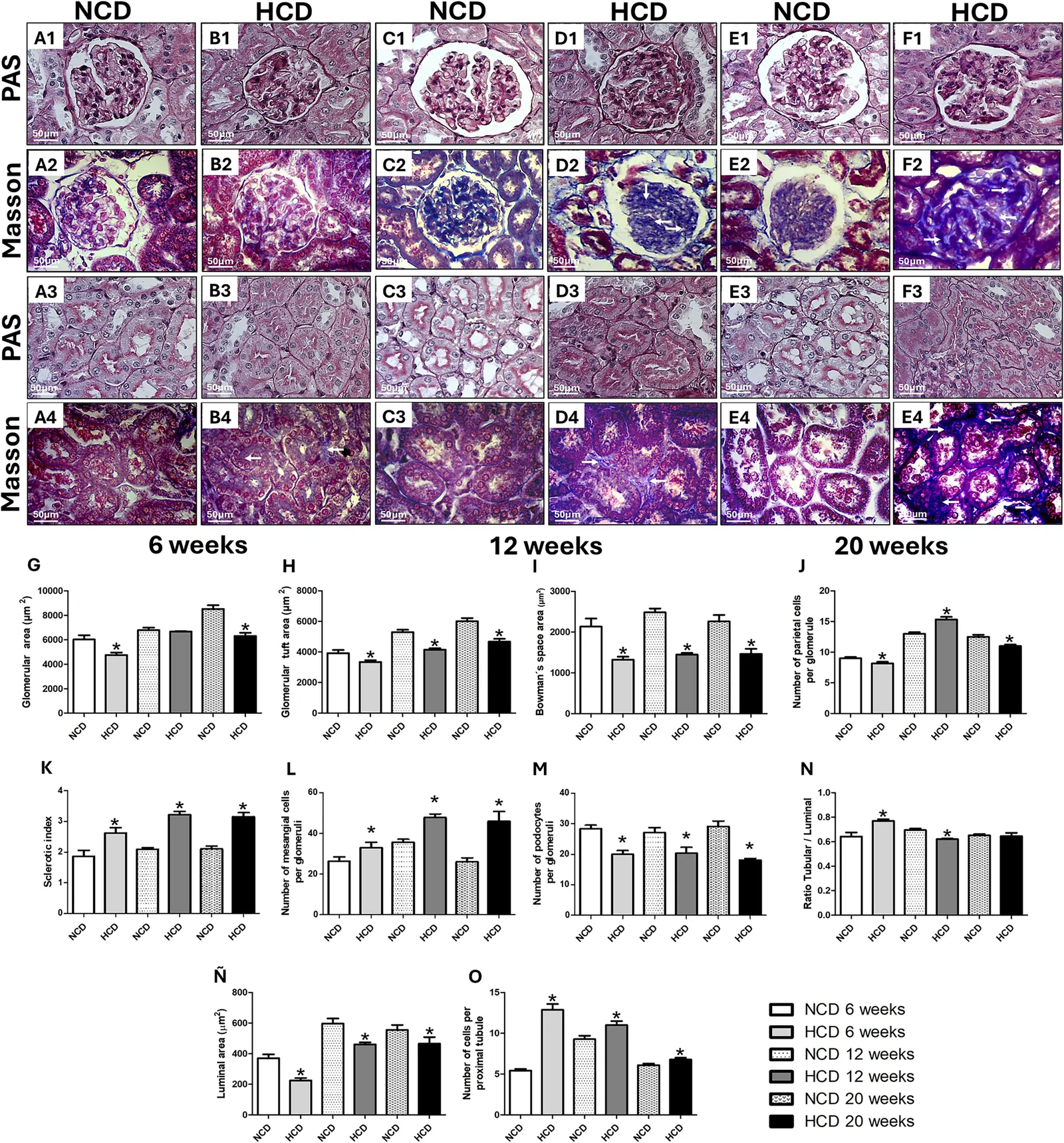
Cortex renal histology. NCD is the standard diet group; HCD is the high-carbohydrate diet group. Glomeruli staining, A1-F1 (PAS stain) and A2-F2 (MT stain). Tubules staining, A3-F3 (PAS stain) and A4-F4 (MT stain). G-M, Glomeruli analysis (*n* = 250); N-O, tubules analysis (*n* = 250). White arrows show collagen deposits. A Two-Way Repeated Measures ANOVA followed by Bonferroni’s post hoc test with a significance level of *P* ≤ 0.05 vs. control was performed. The symbol (*) indicates a significant difference compared to the control group. PAS, periodic acid-Schiff; MT, Masson’s trichrome

Due to metabolic alterations, inflammation, and oxidative stress, the glomerular and tubular structures in the renal cortex were evaluated. We found a significant decrease in the glomerular area over time by 17% (at 6 weeks), and 25.5% (at 20 weeks; F_(5, 245)_ = 3.58, *P* = 0.037; Fig. 4A1-F1 and G), tuft area by 10%, 24%, and 29% (F_(5, 245)_ = 0.683, *P* = 0.5213; Fig. 4H), and Bowman’s space by 37.2%, 48%, and 43% (F_(5, 245)_ = 0.713, *P* = 0.507; Fig. 4I) in the HCD groups compared to the control groups, the analysis identifies time as the primary driver of variance in these datasets. Still, only the decrease in glomerular area showed a significant interaction between diet and time, meaning the effect of the HCD depends on the duration of consumption. The histopathologic analysis also showed that the HCD groups had diminished podocytes by 34.5%, 32.1%, and 43.3% (F_(5, 245)_ = 3.88, *P* = 0.057; Fig. 4M) and parietal cells by 11.1% (at 6 weeks) and 15.4% (at 20 weeks; F_(5, 245)_ = 1.00, *P* = 0.393; Fig. 4J), with an increase in the number of mesangial cells by 20%, 15.8%, and 91% (F_(5, 245)_ = 1.65, *P* = 0.227; Fig. 4L) and the sclerotic index by 47.4%, 63.2%, and 57.9% (F_(5, 245)_ = 1.82, *P* = 0.177; Fig. 4A2-F2, and 4 K). Although the analysis identified HCD consumption as the primary driver of variance in these datasets, time modulated statistical changes and variation in measured parameters; however, the interaction between the two variables was low and non-significant.

The cortical tubule evaluation revealed hypertrophy in the HCD groups throughout the study, characterized by an increased tubular/luminal ratio at 6 weeks by 21% subsequent decrease and stabilization (F_(5, 245)_ = 3.51, *P* = 0.058; Fig. 4A3–F3 and 4 N) and a decreased luminal area over time by 44.7%, 28.8%, and 24.6% (F_(5, 245)_ = 2.51, *P* = 0.117; Fig. 4Ñ). The HCD groups also demonstrated tubular hyperplasia, characterized by increases of 160%, 33.3%, and 40% in the number of tubular cells across all analyzed time points (F_(5, 245)_ = 6.12, *P* = 0.0123; Fig. 4O). In these datasets, the statistical analysis showed that time is the factor that most strongly influences the results, because the effect of a high-carbohydrate diet is not constant but depends on the duration of consumption. Although the interaction between the two variables was low, it was significant only for the number of tubular cells. Masson’s trichrome stain showed progressive collagen over-accumulation in the basement membrane of tubules in the HCD groups (Fig. 4A4 – F4).

### Metabolic syndrome does not generate loss of kidney function but induces renal damage

Interestingly, despite kidney remodeling, clinical biomarkers did not demonstrate kidney disease (Table 3). Azotemia was diminished, with urea levels remaining low from the sixth week onward in the HCD group (43.3%, 27.4%, and 57.1%), and serum creatinine levels decreased at the final evaluation (20 weeks by 23.3%). Two-way repeated-measures ANOVA revealed a highly significant effect of diet on serum urea levels, which were markedly lower in HCD-fed rats across all time points (F_(5, 45)_ = 7.52, *P* < 0.0001). Serum creatinine showed a significant interaction between diet and time, with values decreasing by week 20 in the HCD group (F_(5, 45)_ = 4.86, *P* = 0.0132) compared to the NCD group. Urinary urea excretion increased in concordance with polyuria in the HCD groups. Significant increases were observed in urinary volume (36.8% and 27.6%) and urea excretion in the HCD group starting from week 6 and progressing through week 20 by 25.9%, 28.9%, and 65.4% (F_(5, 45)_ = 24.81, *P* < 0.0001 and F_(5, 45)_ = 4.12, *P* = 0.0245). Meanwhile, creatinine excretion increased only over the first 20 weeks (49.6%); creatinine clearance, a proxy for glomerular filtration rate (GFR), showed a significant increment (34.9% and 58.2%) and effects of both diet and time (F_(5, 45)_ = 3.56, *P* = 0.0384). Rats consuming the HCD exhibited progressive hyperfiltration, reaching significantly higher clearance rates than NCD rats at week 12 (4.95 ± 0.55mL/min) and week 20 (6.2 ± 0.56 mL/min). Despite a trend toward a higher albumin-to-creatinine ratio in HCD groups over time, this difference was not statistically significant (F_(5, 45)_ = 1.03, *P* = 0.4122), and hyperfiltration is likely responsible for this finding (Table 3).

**Table 3:** Renal function panel

	6 weeks			12 weeks			20 weeks		
	NCD *n* = 10		HCD *n* = 10	NCD *n* = 10		HCD *n* = 10	NCD *n* = 10		HCD *n* = 10
Serum									
Creatinine (mg/dL)	0.63 ± 0.02		0.66 ± 0.02	0.67 ± 0.15		0.55 ± 0.13	0.73 ± 0.02		0.56 ± 0.02 ▼
Urea (mg/dL)	46.7 ± 2.22		26.5 ± 1.94▼	46.8 ± 1.52		34 ± 3.27▼	53.1 ± 1.96		22.8 ± 2.76▼
Urine									
Ingest of water (mL/day)	10.7 ± 2.02		9.42 ± 1.58	17.51 ± 1.1		18.12 ± 1.68	22.2 ± 1.40		23.7 ± 2.36
Urinary volume (mL/day)	6.40 ± 0.80		8.10 ± 1.29	17.10 ± 0.49		23.4 ± 1.21▲	19.72 ± 0.96		25.17 ± 0.46▲
Creatinine (mg/24 h)	7.45 ± 1.19		7.74 ± 1.26	10.5 ± 1.30		9.28 ± 1.61	9.54 ± 0.73		14.27 ± 1.07▲
Albumin/creatinine ratio (mg/g)	2.33 ± 0.22		2.41 ± 0.34	3.42 ± 0.45		4.05 ± 0.79	3.88 ± 0.63		4.91 ± 0.81
Urea (mg/24 h)	193 ± 14.6		243 ± 14.8▲	294 ± 11.9		379 ± 16.6▲	211 ± 22.1		349 ± 12.7▲
Creatinine Clearance (mL/min)	2.7 ± 0.28		3.2 ± 0.32	3.67 ± 0.39		4.95 ± 0.55▲	3.92 ± 0.35		6.2 ± 0.56▲

The results shown are the average of 10 different experiments ± SEM. A Two-Way Repeated Measures ANOVA followed by Bonferroni’s post hoc test with a significance level set at *p* ≤ 0.05 was performed. (▲) indicates a significant difference with values above those of the control group. (▼) indicates a significant difference with values below those of the control group

**Fig. 5:**
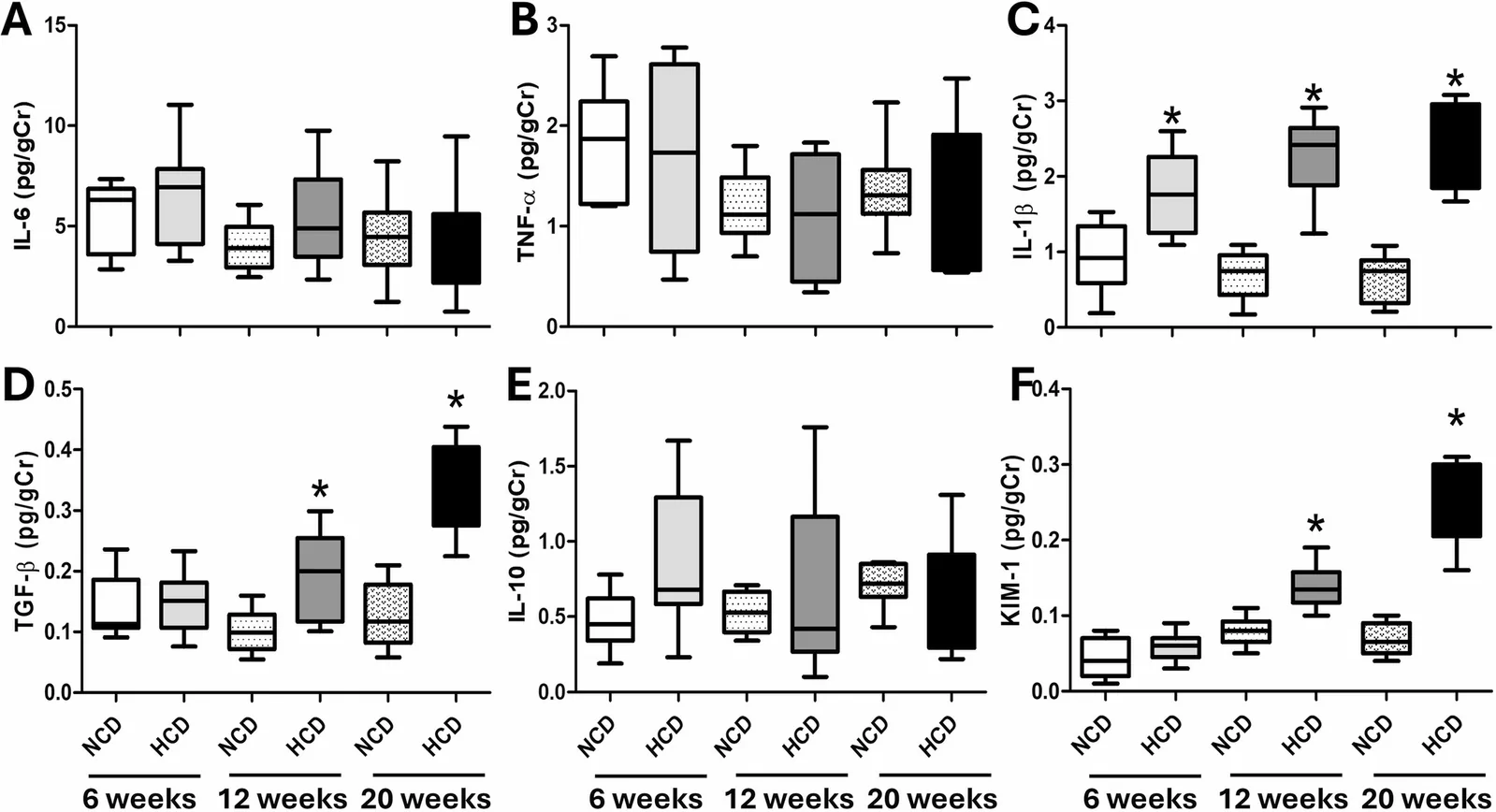
Urinary cytokines and KIM-1. NCD is the standard diet group; HCD is the high-carbohydrate diet group. The results shown are the average of ten different experiments ± SEM. A Two-Way Repeated Measures ANOVA followed by Bonferroni’s post hoc test with a significance level of *P* ≤ 0.05 vs. control was performed. Cytokine and KIM-1 concentrations were normalized to urinary creatinine concentration. The symbol (*) indicates a significant difference compared to the control group. IL-6, interleukin 6; TNF-α, tumor necrosis factor-alpha; IL-1β, interleukin 1 beta; TGF-β, transforming growth factor beta; IL-10, interleukin 10; KIM-1, kidney injury molecule 1

To evaluate the impact of HCD consumption on renal inflammatory status, urinary excretion of key cytokines (IL-6, TNF-α, and IL-1β), normalized to urinary creatinine, was analyzed over 20 weeks. Two-way repeated-measures ANOVA revealed that HCD consumption did not significantly alter urinary excretion of IL-6 (F_(5, 45)_ = 0.137, *P* = 0.873; Fig. 5A) and TNF-α (F_(5, 45)_ = 0.833, *P* = 0.455; Fig. 5B) levels compared with the NCD group, and there was no significant interaction between diet and time. In contrast, a significant main effect of diet was detected for urinary IL-1β levels (F_(5, 45)_ = 12.06, *P* = 0.010; Fig. 5C), indicating higher cytokine excretion in the HCD group by 80% (6 weeks), 145% (12 weeks), and 187% (20 weeks). However, the Bonferroni post hoc analysis indicated that these differences were not sustained throughout the experimental period. These results suggest that while a high-carbohydrate intake may trigger a transient or low-grade increase in specific pro-inflammatory markers, it does not induce a robust or chronic inflammatory response in the urine under the studied conditions.

We also evaluated the progression of renal injury and the compensatory anti-inflammatory response, and analyzed urinary levels of TGF-β, IL-10, and KIM-1. Statistical analysis for TGF-β revealed a significant diet × time interaction (F_(5, 45)_ = 5.43, *P* = 0.018; Fig. 5D) with excretion increasing by 96% and 200% (at 12 and 20 weeks). In contrast, the anti-inflammatory cytokine IL-10 showed no significant changes attributable to diet or time (F_(5, 45)_ = 0.85, *P* = 0.45; Fig. 5E). Regarding tubular integrity, KIM-1 urinary excretion increased by 45% (12 weeks) and 222% (20 weeks). However, the interaction between diet and time was low (F_(5, 45)_ = 1.44, *P* = 0.266; Fig. 5F), suggesting that the high-carbohydrate intervention did not induce significant sustained tubular injury detectable by urinary KIM-1 normalization. Collectively, these data suggest that while HCD triggers a mid-study peak in profibrotic signaling (TGF-β), resulting in low-grade sustained tubular injury, it does not induce an anti-inflammatory shift via IL-10.

## Discussion

In this work, we evaluate the progressive structural changes, damage, and kidney function associated with metaflammation-induced MetS in young-to-adult Wistar rats. As previously informed, we fed rats a high-carbohydrate diet in early life to induce MetS [9, 16, 17]. Adolescent rats were overfed for 6 weeks with an HCD, which led to lipid alterations (hypertriglyceridemia) but not to alterations in glucose homeostasis. This consistent and reproducible pattern likely reflects sustained insulin release-driven lipogenesis [11, 18]. Therefore, *de novo* lipogenesis could be stimulated by insulin resistance via the Sterol-responsive element-binding protein or hyperglycemia via the carbohydrate-responsive element-binding protein, both of which were observed in the HCD groups of adult rats. Then, triglycerides are stored in adipocytes. However, adipocytes that lose insulin sensitivity cannot properly store triglycerides, leading to the release of FFAs, as shown in our results. Therefore, adiposity is not observed, but adiposopathy is developed, supported by the adiponectin/leptin ratio, VAI, and DAI (Table 1) [4, 5, 10]. In adulthood (12- and 20-week groups; 4- and 6-month age), MetS is established by carbohydrate overfeeding, and rats exhibit notable dyslipidemia, dysglycemia, insulin resistance, and severe adipose dysfunction without being overweight (Table 1).

Both animal and human studies have confirmed the association between adiposopathy and metaflammation following excessive caloric intake [19, 20]. A feature of adiposopathy is persistent, low-grade inflammation that fails to resolve and becomes expansive. Expansive inflammation increases plasma levels of cytokines, such as TNF-α, IL-1, and IL-6. It induces energy expenditure similar to that of leptin in metabolically active organs [21, 22]. This is likely one reason why rats fed with HCD do not increase body weight, as both leptin and pro-inflammatory cytokines increase in adult rats (Fig. 1A-C). Notably, serum TGF-β levels increased from early age onward, whereas IL-10 levels did not (Fig. 1D and E). These results suggest MetS generates a systemic imbalance in M1/M2 and Th1/Th2 responses. Elevated concentrations of pro-inflammatory cytokines have been reported in T2D patients and are associated with a Th1 response and a relative suppression of Th2- and Treg-related immunosuppressive cytokines, such as IL-4 and IL-10. The Th1 profile, characterized by suppression of Th2 and Treg cytokines, correlates with the severity of oxidative stress, severe insulin resistance, and the development of micro- and macrovascular complications and renal injury [23, 24]. The statistical analysis suggests that HCD consumption initiates an early profibrotic response (TGF-β at 6 weeks) followed by a broader systemic inflammatory response (IL-6, TNF-α, IL-1β) by 12 weeks. By 20 weeks, the sustained HCD results in significantly reduced anti-inflammatory capacity (IL-10).

Interestingly, serum KIM-1, a biomarker of renal damage, trends to increase in young rats and was significatively augmented in adult rats [25]. KIM-1 is a membrane receptor for T cell immunoglobulin and mucin receptor 1 expressed in the kidneys [26]. KIM-1 functions as a co-stimulatory molecule modulating T cell activation and renal immune signaling [27]. During adolescence (6 weeks), the kidneys are highly active in their development. The initial exposure to experimental conditions—including the physiological transition from childhood to adolescence—can trigger an early inflammatory response. However, at 6 weeks, IL-10, a Th2 immunomodulatory cytokine, is known to modulate and potentially suppress KIM-1 expression. This transient increase in anti-inflammatory defenses likely downregulates KIM-1 shedding into the systemic circulation. KIM-1 is shed into urine after renal tubular injury, but its overexpression is also a marker for the long-term prognosis of CKD [25, 28] Likewise, KIM-1 can promote macrophage production of proinflammatory cytokines, including TNF-α and IL-6 [29]. Besides, KIM-1 is involved in the trafficking of Th1 and Th17 cells during inflammation and kidney injury [23, 26].

For these reasons, we analyzed inflammation in glomeruli and tubules. In the glomeruli of adolescent rats, the immunoreactivity of pro-inflammatory cytokines was higher. However, the same expression dynamics were observed in anti-inflammatory cytokines. Meanwhile, in young adult rats (12 weeks), only IL-6 showed higher immunoreactivity, while the other cytokines maintained the same levels but at reduced levels. Similar immunoreactivity for pro- and anti-inflammatory cytokines was observed in cortical glomeruli of adolescent and adult rats (Fig. 2). Therefore, consumption of an HCD diet and peripheral augmentation of inflammatory cytokines significantly increase glomerular inflammation and early pro-fibrotic changes. In cortical renal tubules, immunoreactivity for pro-inflammatory cytokines increased from adolescence onward, peaking in young adult rats. Meanwhile, IL-10 immunoreactivity exhibited the same expression pattern as pro-inflammatory cytokines, but TGF-β increased only at 12 and 20 weeks of HCD feeding (Fig. 3A-K). This indicates that the tubular inflammatory response is more robust than glomerular immunomodulation, and that the chronicity of carbohydrate dietary overnutrition is definitive in causing tubular damage. The role of inflammation in the pathogenesis and progression of kidney disease has been well recognized. The hypothesis that IL-1β release by monocytes is the master cytokine of inflammation was the starting point for studying renal disease [7, 30]. However, this has been studied only in advanced stages of CKD. Although the initial release of pro-inflammatory cytokines can have favorable effects, persistent inflammation can lead to adverse consequences. The evidence obtained to date indicates that chronic inflammation induces early renal damage, which could favor silent progression of nephropathy; our results show that chronic HCD consumption induces adverse renal conditions that activate local inflammation even before the development of MetS.

Both systemic and intrarenal inflammation and metabolic dysfunction contribute to oxidative stress, which leads to renal injury, nephron dropout, and the onset of CKD. Meanwhile, antioxidative defenses prevent the progression and complications of renal injury [31–33]. Oxidative stress and inflammation are inseparable, major characteristics of kidney damage and drivers of its progression. Our results showed that cortical renal oxidative stress is established simultaneously with MetS (starting at 12 weeks), depleting GSH and GPx activity, the main antioxidant molecules. However, SOD and CAT increase their activity to prevent severe H_2_O_2_ concentration, but with limited success (Table 2). Our results showed that in the “late-stage” defense, GSH is significantly depleted and the 2GSH/GSSG redox index collapses, suggesting that the antioxidant system is unable to maintain a healthy redox balance under chronic HCD consumption. The results also provide evidence that antioxidant kidney defense deteriorates in the early stages of metabolic dysfunction. Evidence for increased renal oxidative stress, accompanied by an adequate antioxidant response, has been reported in cases of renal structural changes with normal function. In the context of progressive kidney disease, findings suggest an interplay among multiple oxidizing mechanisms and decreased antioxidant defenses. The main renal structural modifications associated with oxidative stress are podocyte damage [34, 35], mesangial expansion [30, 36], glomerulosclerosis [37, 38], tubular hypertrophy and hyperplasia [9], and tubulointerstitial fibrosis [30, 31, 36, 38], all of which are observable in adolescent rats before the development of MetS (Fig. 4).

The observed decrease in serum urea and creatinine, alongside increased creatinine clearance from 12 weeks onwards, indicates compensatory hyperfiltration without urinary protein loss (Table 3). The lack of significant albuminuria may reflect a compensatory hyperfiltration phase common in early-stage diabetic or metabolic nephropathy. Mechanistically, this is driven by the early stages of MetS, in which elevated levels of lipoproteins, glucose, and insulin promote the release of pro-inflammatory cytokines, including TNF-α and IL-1β. Consequently, these cytokines promote glomerular hypertrophy and mesangial cell expansion. The significant decrease in glomerular and tuft area, despite hyperfiltration, suggests that the remaining functional nephrons are under extreme hemodynamic stress. At the onset of kidney disease, these adaptations maintain kidney function, preventing proteinuria, glomerular filtration rate (GFR) decline, and macro- and micronutrient loss, as observed in our results. The thickening of the GBM is likely a mechanical response to this pressure, aimed at maintaining the filtration barrier and preventing protein loss, which explains why clinical function appears “normal” despite structural modification. Additionally, hyperleptinemia increases TGF-β levels, leading to profibrotic changes and excessive extracellular matrix deposition. Leptin, via the TGF-2 receptor in mesangial cells, induces expansion and collagen type 1 deposition [39–41]. Studies conducted in Dahl salt-sensitive rats with a dysfunctional leptin receptor derived from zinc-finger nucleases (^SS^LepR mutant strain), which exhibit impaired glucose and insulin resistance, showed significant glomerular injury, including podocyte foot process effacement and lipid droplets, and progression to CKD, from 6 to 18 weeks of age [42]. In the same strain, in prepubertal obesity (4–10 weeks of age). The kidneys exhibited significant glomerular and tubular injury, renal fibrosis, and increased immune cell infiltration, including M1 and M2 macrophages [43], without sex differences. These findings are significant because advanced glycation end-products (hyperglycemia-associated) and lipid peroxides (dyslipidemia- and oxidative stress-associated) cross-link with collagen and other extracellular matrix proteins in GBM, thereby making it stiffer and thicker.

The hypertrophy and hyperplasia observed in cortical tubules serve as mechanisms to handle increased filtrate volume (polyuria). However, persistent local inflammation—evidenced by high IL-6 and TNF-α immunoreactivity in tubules —triggers fibroblasts via TGF-β and IL-1β to deposit collagen. Notably, structural renal modifications, such as tubular-interstitial fibrosis, are associated with oxidative stress and persistent low-grade chronic inflammatory conditions that rapidly activate tubular fibroblasts via TGF-β and IL-1β, secreted by macrophages, leading to extracellular matrix deposition and renal fibrosis [7, 30, 34, 37, 38]. This progressive fibrosis eventually causes hypoxia, linking the metabolic stimulus to chronic structural failure [44, 45]. Therefore, type I and III membrane matrix metalloproteinases are activated to degrade the extracellular matrix, releasing the extracellular portion of KIM-1 into the renal tubular lumen, which is then shed into the urine [44, 45]. KIM-1 immunoreactivity in tubules was detected in the HCD groups, with higher expression at 12 weeks (Fig. 3A-F11 and 3K). In urine, KIM-1, TGF-β, and IL-1β were significatively increased simultaneously, which could be a panel of early biomarkers for nephropathy detection before loss of renal function (Fig. 5). Although urinary KIM-1 has been reported as a predictor of renal injury before changes in GFR were detectable [7, 26, 30], at least to our knowledge, TGF-β and IL-1β had not been reported before in these experimental conditions. By 12 weeks, the rats enter a hyperfiltration stage, during which creatinine clearance increases. This suggests that functional nephrons compensate for structural changes, potentially leading to a temporary “steady state” in which cellular shedding is reduced before terminal fibrosis begins. This is because the chronic metaflammation eventually overrides the 12-week compensatory mechanisms, leading to irreversible fibrosis and the final release of KIM-1 as a marker of established CKD.

In summary, carbohydrate overfeeding from an early age causes metabolic alterations that, in adulthood, contribute to the development of MetS, even in the absence of overweight, but with adiposopathy. As the disease progresses, metaflammation develops, reflected in the serum by elevated levels of inflammatory cytokines. Interestingly, KIM-1 appears in the serum, indicating renal injury. When we investigated the status of renal cortex inflammation, we observed that it begins with the first metabolic disturbances in adolescence, even before oxidative stress, which is associated with MetS in young adult rats. The renal tissue responds to the environment with structural adaptations in both glomeruli and tubules that prevent functional loss, but that slowly progress to kidney disease. A hallmark of this process is the development of fibrosis, characterized by increases in urinary levels of KIM-1, TGF-β, and IL-1β. In conclusion, our results demonstrated that chronic HCD consumption from early life establishes MetS and metaflammation, leading to silent nephropathy. The present experimental study demonstrated that this state is characterized by structural remodeling and profibrotic signaling that occur independently of, and preceding, a decline in standard clinical kidney function markers in Wistar rats. Therefore, serum and urinary cytokine profiles associated with metabolic dysfunction biomarkers may help indicate the onset of kidney disease.

## Data Availability

No datasets were generated or analysed during the current study.
